# Supplementary material for: Revealing the distribution of transmembrane currents along the dendritic tree of a neuron from extracellular recordings
Source: eLife. 2017 Nov 17;6:e29384. doi: 10.7554/eLife.29384 (PMC5716668; doi:10.7554/eLife.29384)
Supplement: Transparent reporting form [file elife-29384-transrepform.pdf]

eLIFE
1st Floor
24 Hills Road
Cambridge CB2 1JP, UK
P 01223 855340
W elifesciences.org
T @elife

eLife's transparent reporting form

We encourage authors to provide detailed information *within their submission* to facilitate the interpretation and replication of experiments. If you have any questions, please contact us: editorial@elifesciences.org.

Sample-size estimation

- You should state whether an appropriate sample size was computed when the study was being designed
- You should state the statistical method of sample size computation and any required assumptions
- If no explicit power analysis was used, you should describe how you decided what sample (replicate) size (number) to use

Please outline where this information can be found within the submission (e.g., page numbers or figure legends), or explain why this information doesn't apply to your submission:

-
-

eLife Sciences Publications, Ltd is a limited liability non-profit non-stock corporation incorporated in the State of Delaware, USA, with company number 5030732, and is registered in the UK with company number FC030576 and branch number BR015634 at the address 1st Floor, 24 Hills Road, Cambridge CB2 1JP | August 2014

In our study we validated the skCSD method with multiple simulated datasets. Our choice of simulated models was to obtain a collection at different levels of complexity, from very simple to biologically plausible models, to investigate how different aspects of neuron morphology and dynamics affect the performance and applicability of the method. Similarly, we considered a range of different putative experimental setups, to see how the experimental circumstances affect the quality of reconstruction.

There are countless possible experimental setups and cell morphologies and we aimed to investigate enough to facilitate understanding of the method while keeping the size of the paper under control.

The following were considered when preparing the simulated data for validation:
- The number and arrangement of electrodes should be relevant to setups currently available on the market. Table 2 (p. 27) shows the numbers of electrodes and their placements for the various morphologies used.
- The complexity of the morphology is reflected by the number of branching points (0 for BS, 1 for Y-shaped, multiple for the Ganglion cell model) and the number of segments is shown in Table 2.
- The current source density pattern in Figure 1 is artificial and the spatial frequencies used were chosen so that at the lowest values it was possible to perform the reconstruction already with 8 electrodes while for the highest frequencies even 128 electrodes were not enough. In the other simulated cases biologically more realistic spatio-temporal membrane current dynamics was used with synaptic activity for which the parameters are summarized in Table 2. and Table 3. We realize it is still far from reality but we fill it sufficiently reflects the spatial resolution of impinging inputs on a cell under various conditions.
- The length of the simulation was connected to the variability of the current source distribution patterns, thus it played a role when selecting optimal reconstruction parameters (basis width and regularization parameter) for the skCSD method.

We provide only one skCSD reconstruction from experimental data since this is the first such data that became available to us. Its goal is to show experimental feasibility of the skCSD method proposed here.

Replicates

- You should report how often each experiment was performed
- The data obtained should be provided and sufficient information should be provided to indicate the number of independent biological and/or technical replicates
- If you encountered any outliers, you should describe how these were handled
- Criteria for exclusion/inclusion of data should be clearly stated
- High-throughput sequence data should be uploaded before submission, with a private link for reviewers provided (these are available from both GEO and ArrayExpress)

Please outline where this information can be found within the submission (e.g., page numbers or figure legends), or explain why this information doesn't apply to your submission:

Fig. 2, 3: reconstruction was performed for 8, 16, 32, 64, 128 electrodes (Fig. legend, not all shown)
Fig. 3: reconstruction was performed for simulation with 100 synaptic activations (Fig. legend, Table 2)
Fig. 4: data described in the manuscript, Fig. legend and Table 2.
Fig. 5: Ground truth data shown in figure and described in the caption. Five random placements of electrodes were used to compare reconstruction from random data with reconstruction from regularly spaced electrodes. Described in the paper (p. 10).
Fig. 6: Since each time step of a simulation gives a different profile of current sources along a cell, multiple profiles were considered over the course of 800 ms of simulation. For illustration of the reconstruction quality, 120 snapshots over this time were used. One snapshot is shown in Fig. 6, the rest in supplementary movie S1 (p. 10, 31).
Fig. 7: The reconstruction was studied for noisy data with signal to noise ratio SNR=16, 4, 1. The reconstructions were done for single realizations of noise and for four different setups (p. 13).
Fig. 8: Reconstruction was done for several different setups, seven are shown here. The reasons for selection, as described in the manuscript, were to show the influence on results of 1. the area covered by the electrodes' grid, and 2. the number of electrodes / electrode density for a given area (p. 13.)
Fig. 9: a single reconstruction for experimental data, described in the manuscript (p. 16).

Statistical reporting

eLife Sciences Publications, Ltd is a limited liability non-profit non-stock corporation incorporated in the State of Delaware, USA, with company number 5030732, and is registered in the UK with company number FC030576 and branch number BR015634 at the address 1st Floor, 24 Hills Road, Cambridge CB2 1JP | August 2014

- Raw data should be presented in figures whenever informative to do so (typically when N per group is less than 10)
- For each experiment, you should identify the statistical tests used, exact values of N, definitions of center, methods of multiple test correction, and dispersion and precision measures (e.g., mean, median, SD, SEM, confidence intervals; and, for the major substantive results, a measure of effect size (e.g., Pearson's r, Cohen's d)
- Report exact p-values wherever possible alongside the summary statistics and 95% confidence intervals. These should be reported for all key questions and not only when the p-value is less than 0.05.

Please outline where this information can be found within the submission (e.g., sections or figure legends), or explain why this information doesn't apply to your submission:

Additional data files ("source data")

SkCSD is a method of data analysis. It is discussed on p. 3-6 and 22-23. Construction of ground truth data and simulations are discussed on p. 23-24. Measuring the quality of reconstruction is discussed on p. 25-26, and parameter selection on p. 26

- • We encourage you to upload relevant additional data files, such as numerical data that are represented as a graph in a figure, or as a summary table
- Include model definition files including the full list of parameters used
- Include code used for data analysis (e.g., R, MatLab)
- Avoid stating that data files are "available upon request"

Please indicate the figures or tables for which source data files have been provided:

Scripts for generating the simulation data used in the paper and the skCSD method implementation are available at the following repository: https://github.com/csdori/skCSD
The scripts generating figures 2-8. presented in the paper together with the necessary data files are uploaded to a Dropbox folder due to the large size (~4GB):
https://www.dropbox.com/sh/azyxpi9ve695sk8/AAAsiWaHK5dWv7Ma3Q6Vuu42a?dl=0
Note that another implementation of the core method is being developed to be integrated in the python-kCSD code https://github.com/asiaszmek/kCSD-python
Once completed it will be merged into the main branch, https://github.com/Neuroinflab/kCSD-python

eLife Sciences Publications, Ltd is a limited liability non-profit non-stock corporation incorporated in the State of Delaware, USA, with company number 5030732, and is registered in the UK with company number FC030576 and branch number BR015634 at the address 1st Floor, 24 Hills Road, Cambridge CB2 1JP | August 2014
